# Hybrid surgery versus anterior cervical discectomy and fusion for multilevel cervical degenerative disc diseases: a meta-analysis

**DOI:** 10.1038/srep13454

**Published:** 2015-08-26

**Authors:** Peng Tian, Xin Fu, Zhi-Jun Li, Xiao-Lei Sun, Xin-Long Ma

**Affiliations:** 1 Department of Orthopaedics, Tianjin Hospital, Tianjin, China; 2 Department of Orthopaedics, Tianjin Medical University General Hospital, Tianjin, China

## Abstract

The objective of this meta-analysis is to compare hybrid surgery (HS) and cervical discectomy and fusion (ACDF) for multilevel cervical degenerative disc diseases (DDD). Systematic searches of all published studies through March 2015 were identified from Cochrane Library, Medline, PubMed, Embase, ScienceDirect, CNKI, WANFANG DATA and CQVIP. Randomized controlled trials (RCTs) and non-RCTs involving HS and ACDF for multilevel DDD were included. All literature was searched and assessed by two independent reviewers according to the standard of Cochrane systematic review. Data of functional and radiological outcomes in two groups were pooled, which was then analyzed by RevMan 5.2 software. One RCT and four non-RCTs encompassing 160 patients met the inclusion criteria. Meta-analysis revealed significant differences in blood loss (p = 0.005), postoperative C2–C7 ROM (p = 0.002), ROM of superior adjacent segment (p < 0.00001) and ROM of inferior adjacent segment (p = 0.0007) between the HS group and the ACDF group. No significant differences were found regarding operation time (p = 0.75), postoperative VAS (p = 0.18) and complications (p = 0.73) between the groups. Hybrid surgery demonstrated excellent clinical efficacy and radiological results. Postoperative C2–C7 ROM was closer to the physiological status. No decrease in the ROM of the adjacent segment was noted in the hybrid surgery group.

Anterior cervical discectomy and fusion (ACDF) has been shown to be a an optimal standard procedure to treat cervical degenerative disc diseases (DDD)^1^. However, many studies have reported that ACDF results in decreased range of motion (ROM) at the fused segments and compensatory increased motion at the adjacent levels, which leads to the acceleration of adjacent segment degeneration (ASD)^2^. Sometimes, symptomatic ASD requires a second surgery.

Artificial cervical disc replacement (A-CDR) serves as an alternative to rigid cervical arthrodesis in the treatment of cervical DDD. The concept is that CDR could stabilize the symptomatic level while preserving some motion, which in theory may reduce the risk of ASD^3^. *In-vitro* biomechanical study also found that CDR restores the functional biomechanical properties of the motion segment, protects neurovascular structures and re-establishes near-normal kinematics to the functional spinal unit^4^. Recently, a few high quality clinical studies have shown that, compared with ACDF, CDR is safe, effective and even advantageous^5^,^6^,^7^.

ACDF, anterior approach direct decompression, was associated with better outcomes than posterior approach (indirect decompression) in the treatment of multilevel cervical spondylotic myelopathy^8^. Traditionally, multilevel ACDF was used to treat cervical spondylotic myelopathy induced by multilevel cervical DDD, resulting in greater loss of mobility in operative levels^9^. Although multilevel cervical DDD could benefit from multilevel CDR, strict indications are narrow for multilevel CDR^10^. Hybrid surgery (HS), combined ACDF and CDR, has advantages in both techniques, especially for multilevel cervical DDD in various degeneration grades^11^,^12^. At present, several published studies have compared HS with ACDF in the treatment of multilevel cervical DDD^13^,^14^,^15^,^16^,^17^. However, controversies still exist between HS and ACDF for treating these patients. Therefore, the current study was conducted to critically review and summarize the literature comparing the results of HS versus ACDF in the treatment of multilevel cervical DDD, in order to identify the better choice.

## Methods

### Search strategy

**Figure 1:**
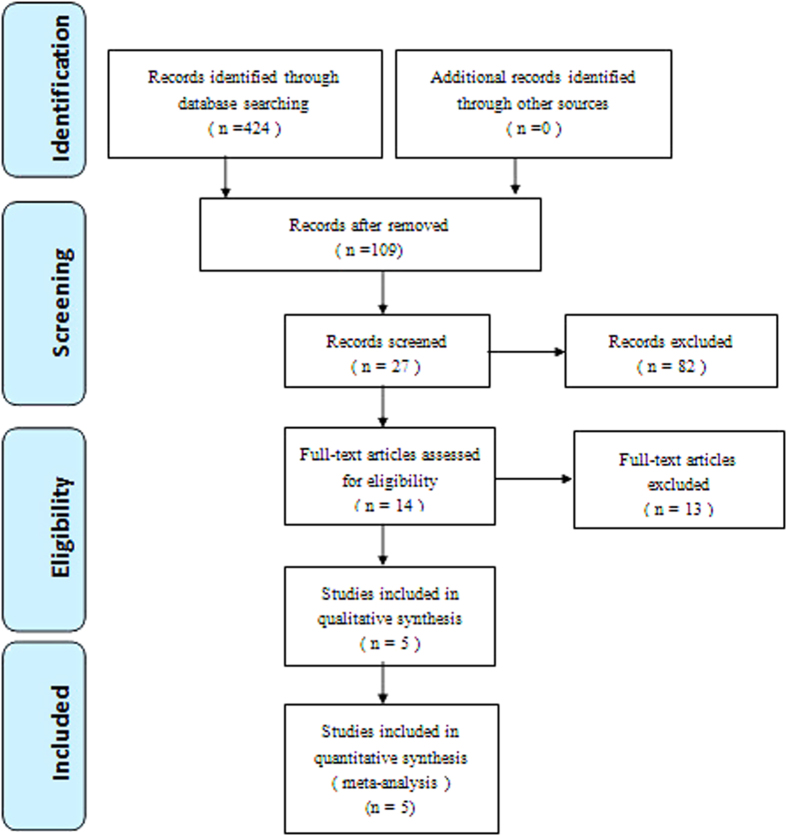
Flowchart of the study selection process.

Electronic databases including Cochrane Library, Medline (1966–2015.3), PubMed (1966–2015.3), Embase (1980–2015.3), ScienceDirect (1985–2015.3), CNKI (1985–2015.3), WANFANG DATA (1985–2015.3) and CQVIP (1985–2015.3) were searched. Gray studies were identified from the reference of included literature. No language was restricted. The search process was conducted as follows in Fig. 1. The key words “hybrid”, “replacement or arthroplasty”, “fusion” and “cervical” were used in combination with the Boolean operators AND or OR.

### Inclusion criteria

Studies were considered eligible for inclusion if they met the following criteria:

Study design: Interventional studies (RCTs or non-RCTs).

Population: Patients with multilevel cervical DDD.

Intervention group: HS (ACDF+CDR).

Control group: ACDF.

Outcomes: Reported at least one of the following: blood loss, operative time, subjective pain perception, neck disability index (NDI), Japanese Orthopaedic Association (JOA) scale, C2–C7 range of motion (ROM), adjacent level ROM and complications.

### Exclusive criteria

Patients were excluded from the meta-analysis if they had neoplastic etiology (i.e., metastasis or myeloma), infection, traumatic fracture, spondylolisthesis, serious osteoporosis, metal sensitivity or mental diseases.

### Selection criteria

Two reviewers independently screened the titles and abstracts for the eligibility criteria. Subsequently, the full text of the studies that potentially met the inclusion criteria were read, and the literature was reviewed to determine the final inclusion. Disagreement was resolved by consulting a third reviewer.

### Quality assessment

Quality assessment for randomized trial was conducted according to a modification of the generic evaluation tool used by the Cochrane Bone, Joint and Muscle Trauma Group^18^ and index for non-randomized studies (MINORS) form for non-randomized clinical trials^19^. The methodological quality of each trial was scored from 0 to 24. Disagreements were resolved by consensus or consultation with the senior reviewer.

### Data extraction

Two researchers independently extracted the data from the included literature. In the case of incomplete data, the corresponding author was consulted for details. The following information was extracted: First author name, year of publication, intervening measures, comparable baseline, sample size and outcome measures. Other relevant parameters were also extracted from individual studies.

### Data analysis and statistical methods

Pooling of data was analyzed by RevMan 5.1 (The Cochrane Collaboration, Oxford, United Kingdom). Heterogeneity was estimated depending on the value of P and I^2^ using the standard chi-square test. When I^2^ > 50%, P < 0.1 was considered to be significant heterogeneity. Therefore, a random-effects model was applied for data analysis. A fixed-effects model was used when no significant heterogeneity was found. In case of significant heterogeneity, subgroup analysis was performed to investigate sources. For continuous outcomes, mean differences (MDs) and 95% confidence intervals (CIs) were presented. Risk difference (RD) and 95% CIs were calculated for dichotomous data.

## Results

### Literature search

A total of 424 potential studies were identified with the first search strategy. Of these, 419 reports were excluded according to the eligibility criteria. No additional studies were obtained after the reference review. Ultimately, four non-RCTs and one RCT^13^,^14^,^15^,^16^,^17^ were eligible for data extraction and meta-analysis, as indicated by the flowchart in Fig. 1. These studies involved a total of 74 patients in the HS group and 86 patients in the ACDF group.

### Study characteristics

The main characteristics of the included studies are reported in Table 1. Statistically similar baseline characteristics were observed between the HS and ACDF groups. All studies had small sample sizes, from 14 to 48 patients. The number of operative levels was two or three. The design of CDR varied among different studies. All studies except one were followed up more than 18 months^15^.

### Risk of bias assessment

**Figure 2:**
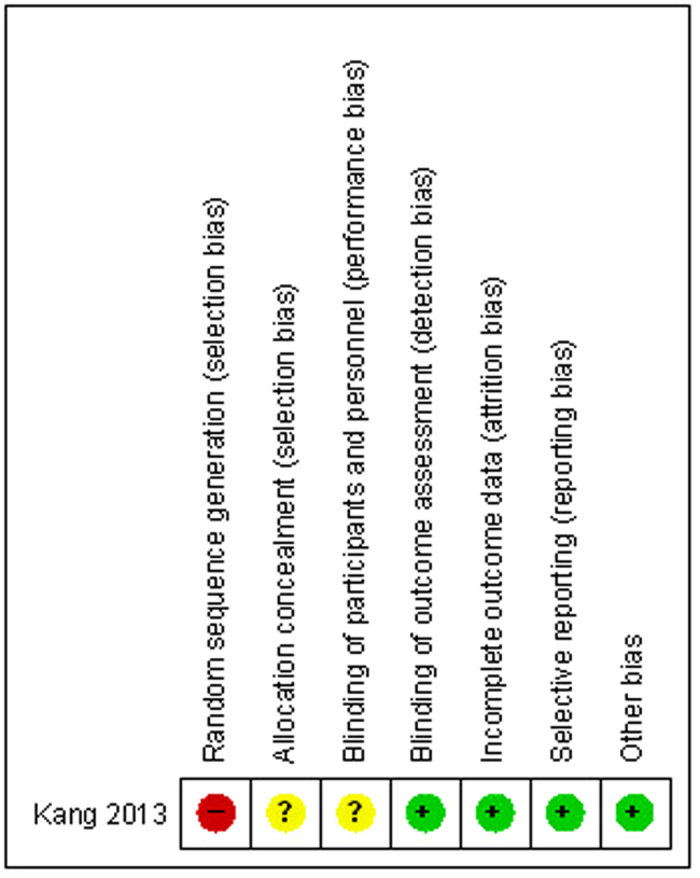
The summary of bias risk of randomized controlled trials.

The quality of the included studies was assessed according to the Cochrane Handbook for Systematic Review of Interventions. Only one RCT^17^ met the inclusion criteria. Randomization was conducted using the odd or even hospital number, which showed a high risk of selection bias; adequate concealment of allocation was unclear for RCT. For the non-RCTs^13^,^14^,^15^,^16^, the MINORS score was 15-17 for the retrospective controlled trials. The methodological quality assessment is illustrated in Fig. 2 (RCT) and Table 2 (non-RCT).

### Outcomes for meta-analysis

#### Operation time

**Figure 3:**
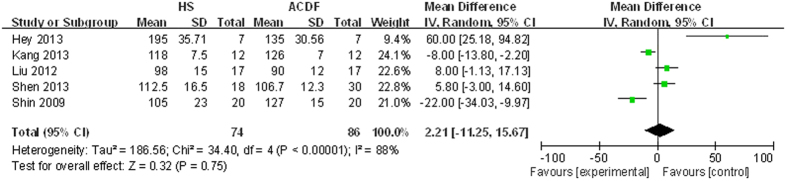
Forest plot of operation time between two groups.

Details regarding operation time were available in five studies^13^,^14^,^15^,^16^,^17^. There was significant heterogeneity (*χ*^2^ = 34.40, df = 4, I^2^ = 88%, P < 0.00001); therefore, a random-model was performed. Pooling results demonstrated that operation time made no significant difference between the two groups (MD = 2.21, 95% CI: −11.25 to 15.67, P = 0.75; Fig. 3).

#### Blood loss

**Figure 4:**
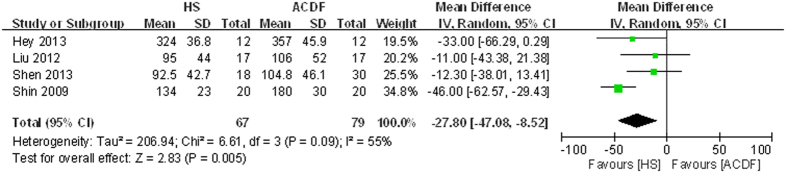
Forest plot of blood loss between two groups.

Four articles reported blood loss^13^,^14^,^15^,^16^. Significant heterogeneity was found, so a random-model was used (*χ*^2^ = 6.61, df = 3, I^2^ = 55%, P = 0.09). There was significance between HS groups and ACDF groups regarding blood loss (MD = −27.80, 95% CI: −47.08 to −8.52, P = 0.005; Fig. 4).

#### Preoperative and postoperative visual analog scale (VAS)

Two studies showed preoperative and postoperative 24 months VAS^14^,^17^. For preoperative VAS, there was no significant heterogeneity, so a fixed-model was performed. No significant difference was found between HS groups and ACDF groups (MD = −0.10, 95% CI: −0.85 to 0.65, P = 0.79; Table 3). For postoperative 24 months VAS, there was significant heterogeneity; thus, a random-model was performed. No significant difference was found between HS groups and ACDF groups (MD = −1.00, 95% CI: −2.46 to 0.47, P = 0.18; Table 3).

#### Preoperative and postoperative C2–C7 ROM

Relevant data regarding the preoperative C2–C7 ROM were documented in four articles^14^,^15^,^16^,^17^. No significant heterogeneity was found, so a fixed-model was applied. There was significant difference between HS groups and ACDF groups (MD = 0.51, 95% CI: −3.73 to 4.74, P = 0.81; Table 3). Postoperative 24 months C2–C7 ROM were documented in two articles^13^,^17^. No significant heterogeneity was found; thus, a fixed-model was applied. There was significant difference between HS groups and ACDF groups (MD = 9.50, 95% CI: 1.44 to 15.40, P = 0.002; Table 3).

#### Preoperative and postoperative ROM of the superior adjacent segment

Four articles reported the preoperative ROM of the superior adjacent segment^14^,^15^,^16^,^17^. A fixed-model was used due to low-significance heterogeneity and showed no significant difference between groups (MD = −0.46, 95% CI: −2.15 to 1.23, P = 0.60; Table 3). Two articles reported the postoperative 24 months ROM of the superior adjacent segment^13^,^17^. A random-model was used due to high-significance heterogeneity and showed a significant difference between groups (MD = −5.24, 95% CI: −7.57 to −2.92, P < 0.00001; Table 3).

#### Preoperative and postoperative ROM of the inferior adjacent segment

Preoperative ROM of the inferior adjacent segment was provided in four trials^14^,^15^,^16^,^17^. No significant heterogeneity was showed between pooling results; thus, a fixed-model was performed. There was no significant difference between groups (MD = −0.45, 95% CI: −1.78 to 0.87, P = 0.50; Table 3). Postoperative 24 months ROM of the inferior adjacent segment was provided in two trials^13^,^17^. No significant heterogeneity was shown between pooling results; thus, a fixed-model was performed. There was significant difference between groups (MD = −4.62, 95% CI: −7.28 to −1.96, P = 0.0007; Table 3).

#### Complications

**Figure 5:**
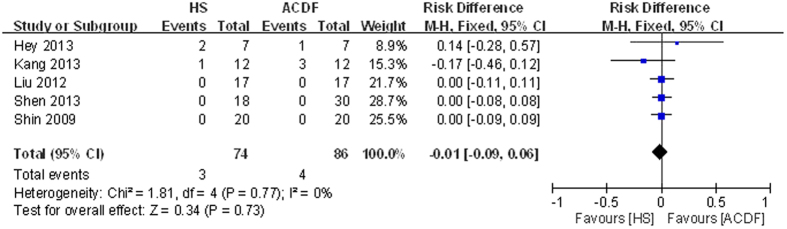
Forest plot of complications between two groups.

Data regarding complications were provided in all studies^13^,^14^,^15^,^16^,^17^. No significant heterogeneity was shown between pooling results; thus, a fixed-model was performed. There was no significant difference between groups (RD = −0.01, 95% CI: −0.09 to 0.06, P = 0.73; Fig. 5).

#### Other outcomes

Neck Disability Index (NDI) scores and Japanese orthopaedic association (JOA) scores were used to assess functional recovery in most studies. However, limited data could not be extracted for meta analysis, as shown in Table 4. Only one study reported better recovery of NDI at 24 months postoperatively for the HS group^14^.

## Discussion

The most important findings of the present study were that HS reduces blood loss, postoperative C2–C7 ROM was close to the physiological level, and no decrease in the ROM of the adjacent segment was noted in the HS group. Although most included studies reported consistent results, more consideration should be given when we interpret these results.

For published studies with small samples, we searched and included all the RCT and non-RCT. Four non-RCTs^13^,^14^,^15^,^16^ and one RCT^17^ met the inclusion criteria in the meta-analysis. There were biases for randomization and concealment of allocation in the RCT. Quality-assessment scores of non-RCTs ranged from 15 to 17. No prospective calculation of the sample size was described in non-RCTs. In addition, the assessment of the study endpoints was biased. All these shortcomings weaken the level of evidence.

In 2014, Jia *et al.* performed a systematic review of biomechanical and clinical evidence of HS for multilevel cervical DDD^20^. Although their results are consistent with our meta-analysis, they did not extract data for further quantitative analysis. To our knowledge, this is the first quantitative meta-analysis to evaluate HS for multilevel cervical DDD by only including studies that had appropriate control and study groups.

Previous meta-analysis^21^,^22^,^23^,^24^ reported that ACDF was associated with shorter operative times and less blood loss in single level. In our meta-analysis, HS reduced blood loss and did not increase operation time for multilevel cervical DDD. Decreased blood loss and operation time for CDR are attributable to the surgeons experienced with this technology. Therefore, CDR is considered less invasive.

In our meta-analysis, preoperative VAS scores were not significantly different between the two groups. Postoperative 24 months VAS scores significantly improved in each group, without significant differences between the two groups. Although the functional assessment did not perform a quantitative meta-analysis due to limited data, most of the included studies reported that there was no significant difference between the two groups. These results suggest that both procedures effectively reduce patients’ pain and improve function.

HS aims to keep the kinetics of the adjacent segment to the greatest extent avoiding ASD. Present meta-analysis showed that HS improved postoperative 24 months C2–C7 ROM and decreased superior and inferior adjacent segment ROM compared with ACDF. These results are consistent with previous biomechanical studies. Cervical motion after HS is closer to the physiological kinetics than after ACDF.

In all, 7 complications were reported in the two groups, including 1 dysphasia, 1 heterotopic ossification, and 1 residual limb symptom for the HS group. For the ACDF group, the corresponding complications were 1, 1 and 2, respectively. Meta-analysis showed that there was no significant difference between the two groups. This result suggests that both the treatments are safe in short-term follow-up. Recently, a meta-analysis of 4 included RCTs with 4–7 years of follow-up showed that CDR results in better clinical and radiographic outcomes^7^. Complications may increase in patients with middle- or long-term follow-up.

Several potential limitations should be acknowledged in the present meta-analysis: 1) Only one RCT and four non-RCTs were identified, and the sample sizes of the included studies were relatively small; 2) There were some methodological weaknesses in the included RCT and non-RCTs; 3) Because some data were incomplete, we failed to conduct meta-analysis such as functional score; 4) Follow-up was relatively short and may lead to underestimating complications.

## Conclusion

Hybrid surgery demonstrated excellent clinical efficacy and radiological results. Postoperative C2–C7 ROM was closer to the physiological status. No decrease in the ROM of the adjacent segment was noted in the hybrid surgery group. Large sample sizes, long-term follow up, and well designed studies are needed in the future.

## Additional Information

**How to cite this article**: Tian, P. *et al.* Hybrid surgery versus anterior cervical discectomy and fusion for multilevel cervical degenerative disc diseases: a meta-analysis. *Sci. Rep.* **5**, 13454; doi: 10.1038/srep13454 (2015).

## Figures and Tables

**Table 1:** Characteristics of included studies.

Study	Group	Simple size	Age (Y)	Gender (M/F)	No. of level (Two/Three)	Devices information	Follow-up (months)
Hey 2013	HS	7	51	3/4	4/3	ProDisc-C	48
	ACDF	7	48	4/3	4/3	cage	
Kang 2013	HS	12	53.6	8/4	0/12	ProDisc-C	24–48
	ACDF	12	55.3	7/5	0/12	cage/Zero-plate	
Liu 2012	HS	17	53.7	13/4	12/0	NS	6
	ACDF	17	56.4	14/3	12/0		
Shen 2013	HS	18	54.2	11/7	18/0	Bryan	18–34
	ACDF	30	54.9	19/11	30/0	cage	
Shin 2009	HS	20	48	10/10	20/0	Mobi-C	>24
	ACDF	20	45.7	12/8	20/0	cage	

HS: hybrid surgery, ACDF: anterior cervical discectomy and fusion, NS: not state, M: male, F: female, Y: years.

**Table 2:** Quality assessment for non-randomized trials.

Quality assessment for non-randomized trials	Hey 2013	Liu 2012	Shen 2013	Shin 2009
A clearly stated aim	2	2	2	2
Inclusion of consecutive patients	2	1	1	1
Prospective data collection	2	2	2	2
Endpoints appropriate to the aim of the study	1	1	1	1
Unbiased assessment of the study endpoint	0	0	0	1
A follow-up period appropriate to the aims of study	2	2	2	2
Less than 5% loss to follow-up	2	2	2	2
Prospective calculation of the sample size	0	0	0	0
An adequate control group	2	1	2	1
Contemporary groups	1	0	1	1
Baseline equivalence of groups	2	2	2	2
Adequate statistical analyses	2	2	2	1
Total score	16	15	17	15

**Table 3:** Meta-analysis results.

Outcome	Studies	Groups (HS/ACDF)	Overall effect		p-Value	Heterogeneity	
			Effect estimate	95% CI		I^2^(%)	p-Value
Operation time	5	74/86	2.21	−11.25, 15.67	0.75	88	<0.00001
Blood loss	4	67/79	−27.80	−47.08, −8.52	0.005	55	0.09
Complications	5	74/86	−0.01	−0.09, 0.06	0.73	0	0.77
VAS							
Preoperative	2	32/32	−0.10	−0.85, 0.65	0.79	0	1.00
Postoperative 24 months	2	32/32	−1.00	−2.46, 0.47	0.18	75	0.05
C2–C7 ROM							
Preoperative	4	67/79	0.51	−3.73, 4.74	0.81	0	0.99
Postoperative 24 months	2	32/32	9.50	1.44, 15.40	0.002	0	0.92
Superior adjacent level ROM							
Preoperative	4	67/79	−0.46	−2.15, 1.23	0.60	0	0.58
Postoperative 24 months	2	32/32	−5.24	−7.57, −2.92	<0.00001	54	0.14
Inferior adjacent level ROM							
Preoperative	4	67/79	−0.45	−1.78, 0.87	0.50	0	0.41
Postoperative 24 months	2	32/32	−4.62	−7.28, −1.96	0.0007	0	0.59

HS: hybrid surgery, ACDF: anterior cervical discectomy and fusion, ROM: range of motion, CI: confidence interval.

**Table 4:** Functional assessment of included studies.

	Follow up (m)	HS	ACDF	p values
NDI				
Hey 2013	24	17.8 ± 4.197	20 ± 1.44	0.506
Kang 2013	24	16%	19%	>0.05
Liu 2012	6	20.0%	22.2%	>0.05
Shen 2013	24.1(18–34)	22.5 ± 5.1	23.1 ± 5.2	>0.05
Shin 2009	24	19%	24%	>0.05
JOA				
Liu 2012	6	16.4 ± 0.7	16.2 ± 0.7	>0.05
Shen 2013	24.1(18–34)	16.5 ± 0.9	16.3 ± 0.8	>0.05

NDI: Neck Disability Index, JOA: Japanese orthopaedic association, HS: hybrid surgery, ACDF: anterior cervical discectomy and fusion.
